# Clinical Significance of Preoperative Serum CEA, CA125, and CA19-9 Levels in Predicting the Resectability of Cholangiocarcinoma

**DOI:** 10.1155/2019/6016931

**Published:** 2019-02-04

**Authors:** Tianyi Fang, Hao Wang, Yufu Wang, Xuan Lin, Yunfu Cui, Zhidong Wang

**Affiliations:** ^1^Department of Hepatopancreatobiliary Surgery, Second Affiliated Hospital of Harbin Medical University, Harbin Medical University, Harbin, China; ^2^Department of Gastrointestinal Surgery, Harbin Medical University Cancer Hospital, Harbin Medical University, Harbin, China

## Abstract

To explore the clinical significance of preoperative serum CEA, CA125, and CA19-9 levels in predicting the resectability of cholangiocarcinoma. Patients with cholangiocarcinoma diagnosed by radiologic examination and admitted to the Second Affiliated Hospital of Harbin Medical University from September 1, 2011, to November 30, 2017, were retrospectively included. The relationship between the preoperative serum CEA, CA125, and CA19-9 levels and the resectability of cholangiocarcinoma was analyzed by receiver operating characteristic (ROC) curve, as well as the best cut-off point. A total of 112 met the inclusion criteria. In 50 patients with radical surgeries, the levels of preoperative serums CEA, CA125, and CA19-9 were 5.0 ± 13.9 ng/mL, 15.3 ± 11.8 U/mL, and 257.5 ± 325.6 U/mL, respectively, which were lower than those in patients with unresectable tumor. Based on the ROC curve, the ideal CA19-9 cut-off value was determined to be 1064.1 U/mL in prediction of resectability, with a sensitivity of 53.2%, a specificity of 94.0%, and the area under the ROC curve of 0.73 (*P* < 0.05). The cut-off value of CA125 was 17.8 U/mL with a sensitivity of 72.6%, a specificity of 78.0%, and the area under the ROC curve of 0.81 (*P* < 0.05). The cut-off value of CEA was 2.6 ng/mL with a sensitivity of 79.0%, a specificity of 48.0%, and the area under the ROC curve of 0.66 (*P* < 0.05). In addition to this, we found that using the combination of three tumor markers could improve the value in predicting resectability of cholangiocarcinoma. In summary, this study suggested that the preoperative serum CEA, CA125, and CA19-9 levels can help predict the resectability of cholangiocarcinoma.

## 1. Introduction

Cholangiocarcinoma is the most common primary tumor of the biliary tract, with a poor prognosis at the advanced stages [1–3]. The morbidity and mortality of cholangiocarcinoma have increased over the past 40 years, especially in Asia [4]. Currently, radical resection is accepted widely as one of the preferred treatment options for cholangiocarcinoma [5, 6]. However, cholangiocarcinoma is closely associated with adjacent structures, such as the portal vein and hepatic artery [7], and it is characterized by an infiltrative growth [8, 9]. In addition, due to its insidious onset and high malignancy, it is often found in advanced stages at diagnosis [10]. Therefore, the surgical resection rate of cholangiocarcinoma is low [11, 12]. It is of increasing clinical importance to evaluate the resectability of the tumor before operation.

Currently, the assessment of resectability of cholangiocarcinoma is based on a combination of clinical, radiological, and biochemical approaches [13, 14]. Cholangiocarcinoma grows along the wall of the bile duct or the connective tissue around the bile duct, forming no nodule or mass in many cases, thus displaying no mass shadow in radiological examinations [15, 16], whereas surgical exploration is invasive and may result in a huge financial burden on patients.

There is no biomarker currently available for the resectability of cholangiocarcinoma that is sufficiently sensitive and specific. As key markers for the diagnosis of gastrointestinal malignancies and predicting their prognosis, carcinoembryonic antigen (CEA), carbohydrate antigen (CA)125, and CA19-9 have also been widely used to predict the resectability of tumors [17–19]. At present, they are also important markers in the diagnosis of cholangiocarcinoma. It was reported that preoperative serum CA19-9 level was positively associated with tumor stage. However, the increase in CA19-9 may be caused by cholangitis or obstructive jaundice. Thus, it may not be accurate to use CA19-9 alone in clinical practice. Mucins have been associated with human malignant tumors. CA125 is currently considered to be MUC16, and its amino acid sequence has some properties of mucin molecules, which may have better clinical application in adenocarcinoma [20]. CEA levels are not related to serum bilirubin levels and may be effective in predicting surgical resection rates. In general, it remains unclear whether their expression levels are valuable in determining the resectability of cholangiocarcinoma. In this study, we retrospectively analyzed the preoperative serum CEA, CA125, and CA19-9 levels in 112 patients with cholangiocarcinoma who were treated in our center from 2011 to 2017 and explored their clinical value in determining the resectability of cholangiocarcinoma.

## 2. Materials and Methods

We retrospectively analyzed clinically diagnosed or pathologically confirmed cholangiocarcinoma patients who had been admitted to the Second Affiliated Hospital of Harbin Medical University from September 1, 2011, to November 30, 2017. The clinical diagnosis was mainly based on clinical symptoms, imaging findings [computed tomography (CT), ultrasonography, magnetic resonance imaging, positron emission tomography–CT, and endoscopic ultrasonography], and tumor markers including CEA, CA125, and CA19-9 [21]. The patients were staged according to the American Joint Committee on Cancer (AJCC) Staging System. Tumor resectability was confirmed by intraoperative exploration; all patients were from more than two chief physicians to determine whether to perform radical surgery. In addition, a tumor was confirmed to be unresectable if radiological examination revealed the presence of hepatic metastasis or other distant metastasis. In order to make sure the decision is uniformed, most of the patients were selected from nearly three years and treated by three chief physicians. Patients with incomplete clinical data were excluded.

### 2.1. Determination of Serum CA19-9, CA125, and CEA Levels

Before treatment, 5 mL peripheral blood was extracted from the peripheral vein, and plasma and albumin were isolated by centrifugation at 2000 × *g* for 15 min. The CA125 and CA19-9 levels were determined by radioimmunoassay [22, 23], with a normal upper limit of 35 U/mL and 37 U/mL, respectively. The CEA level was determined by ELISA [24], with a normal upper limit of 5 ng/mL.

### 2.2. Statistical Analysis

Statistical analysis was performed using SPSS 22.0. Numerical data were presented as the mean ± standard deviation. A two-tailed *P* value < 0.05 was considered statistically significant. The optimal cut-offs for CEA, CA125, and CA19-9 in determining the resectability were analyzed using the receiver operating characteristic (ROC) curves.

## 3. Results

In total, 112 cholangiocarcinoma patients (66 men and 46 women; male/female ratio 1.43; average age 62.5 years) were retrieved. The disease was pathologically confirmed in 72 cases and clinically diagnosed in 40 cases. Although pathological diagnosis was more reliable, clinical diagnosis was acceptable based on the patients' symptoms and accessory examinations.

### 3.1. General Clinical Features of Cholangiocarcinoma Patients

Cholangiocarcinoma was resectable in 50 cases (44.6%) and unresectable in 62 cases (55.4%). The lesions were located in hepatic segments in 16 cases, at the hepatic hilum in 35 cases, and in the distal bile duct in 61 cases. There were 50 cases of radical resection and 22 cases of palliative resection, which were also histologically classified as highly (*n* = 24), moderately (*n* = 28), or poorly (*n* = 20) differentiated. The rest of the patients were treated by endoscopic or ultrasound intervention, so there was no pathological diagnosis. The AJCC staging results were as follows: two stage I, resection rate 100%; 44 stage II, resection rate 93.2%; 24 stage III, resection rate 29.2%; and 42 stage IV, all of which were unresectable. The sizes of resectable tumor tissue were determined consistently by a trained pathologist, and those of the unresectable group were determined consistently by a trained radiologist. The average tumor diameter was 2.3 ± 0.9 cm in the resectable group, which was significantly smaller than that of the unresectable group (4.5 ± 1.6 cm, *P* < 0.05, Table 1).

### 3.2. Serum CEA, CA125, and CA19-9 Levels in Determining Cholangiocarcinoma Resectability

Table 2 shows the multivariate logistic regression models for predicting the resectability of cholangiocarcinoma. Multivariate logistic regression analysis for predicting the resectability of cholangiocarcinoma showed that the serum levels of CEA, CA125, and CA19-9 had a better predictive value in radical resection.

**Figure 1:**
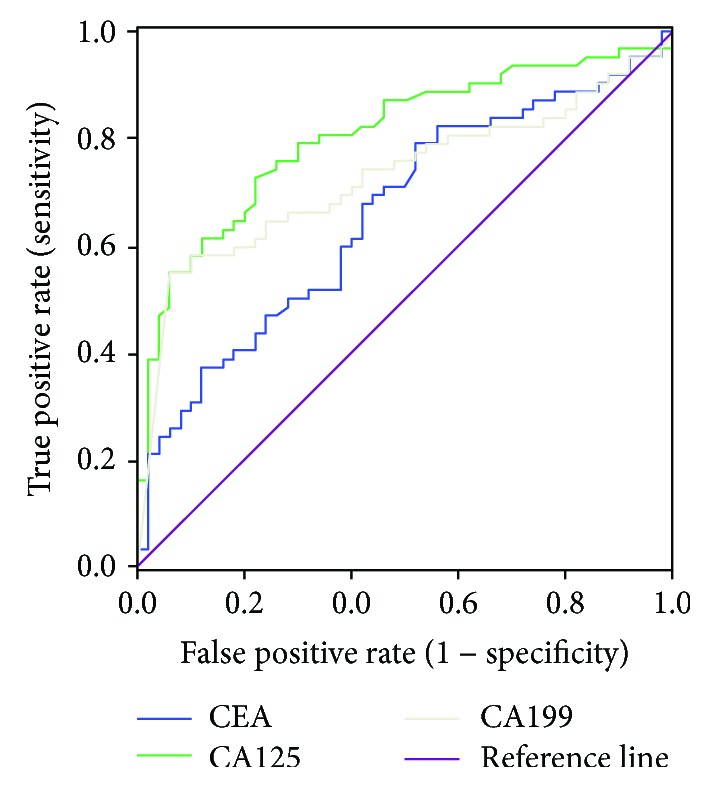
ROC curves for serum CEA, CA125, and CA19-9 levels in the determination of cholangiocarcinoma resectability. The AUC is 0.66 for CEA, 0.81 for CA125, and 0.73 for CA19-9.

Serum CEA, CA125, and CA19-9 levels in the resectable group (*n* = 50) were 5.0 ± 13.9 ng/mL, 15.3 ± 11.8 U/mL, and 257.5 ± 325.6 U/mL, respectively, which were significantly lower than those in the unresectable group (19.1 ± 69.2 ng/mL, 48.8 ± 58.7 U/mL, and 730.1 ± 527.5 U/mL, respectively). According to the results of the ROC curve analysis, the optimal cut-offs for determining the resectability of cholangiocarcinoma were as follows (Figure 1). When CA19-9 was 1064.1 U/mL, it had a sensitivity of 53.2%, specificity of 94%, positive predictive value (PPV) of 80.8%, and negative predictive value (NPV) of 0.73, and the area under the ROC curve (AUC) was 0.73 [95% confidence interval (CI): 0.63–0.82]. When CA125 was 17.8 U/mL, it had a sensitivity of 72.6%, specificity of 78.0%, PPV of 76.7%, and NPV of 74.0%, and the AUC was 0.81 (95% CI: 0.72–0.89). When CEA was 17.8 U/mL, it had a sensitivity of 79.0%, specificity of 48.0%, PPV of 75.5%, and NPV of 53.0%, and the AUC was 0.66 (95% CI: 0.56–0.76).

**Figure 2:**
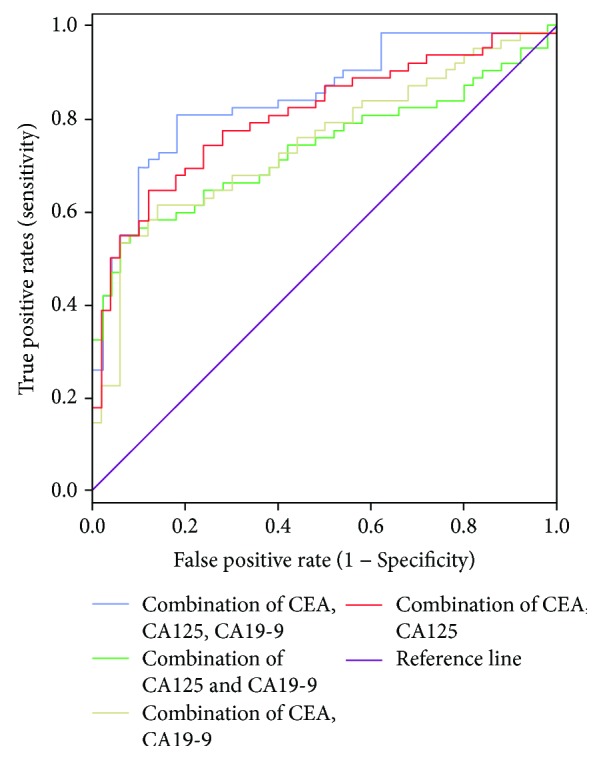
ROC curves for the combination of serum CEA, CA125, and CA19-9 levels in the determination of cholangiocarcinoma resectability. For the combination of CEA and CA125, the AUC is 0.81; for the combination of CEA and CA19-9, the AUC is 0.75; for the combination of CEA, CA125 and CA19-9, the AUC is 0.74; and for the combination of CEA, CA125, and CA19-9, the AUC is 0.87.

By logistic regression analysis, we found that using the combination of three tumor markers could improve the value of predicting resectability of cholangiocarcinoma (Figure 2). The results of ROC curve analysis showed that when we used the combination of CEA and CA125, the AUC was 0.81 (95% CI: 0.89–0.73); when we used the combination of CEA and CA19-9, the AUC was 0.75 (95% CI: 0.84–0.66); when we used the combination of CA125 and CA19-9, the AUC was 0.74 (95% CI: 0.83–0.64); and when we used the combination of CEA, CA125, and CA19-9, the AUC was 0.87 (95% CI: 0.92–0.78). (*P* < 0.05).

### 3.3. Value of Serum Total Bilirubin in Determining Cholangiocarcinoma Resectability

**Figure 3:**
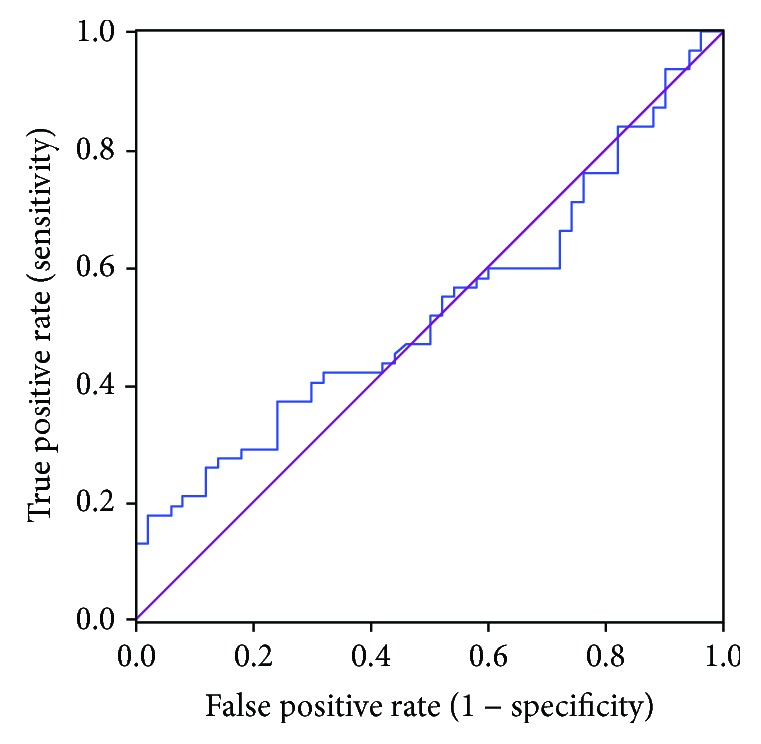
The ROC curve of serum total bilirubin level in the determination of cholangiocarcinoma resectability. The blue line is the total bilirubin, and the purple line is the reference line. The AUC of total bilirubin is 0.54.

ROC curve analysis showed that the AUC was 0.54 (95% CI: 0.43–0.65), suggesting that serum total bilirubin had an extremely low accuracy in predicting the resectability of cholangiocarcinoma (Figure 3).

## 4. Discussion

Surgery remains the treatment of choice for cholangiocarcinoma [25, 26]. Radical resection can be achieved if the patient's general condition can tolerate the operation, and there is no distant metastasis [27–30]. Despite rapid advances in surgical techniques and postoperative management, the overall resection rate of cholangiocarcinoma remains low [1, 3, 31], and the incidence of postoperative fatal complications is still high [26, 32, 33]. Therefore, accurate and reliable determination of the resectability of cholangiocarcinoma before surgery is important. CT is the most common examination for determining the resectability of cholangiocarcinoma [34, 35]. However, it is often unable to detect occult metastatic lesions in the liver or abdominal cavity and may miss vascular invasion, resulting in unnecessary surgical trauma and waste of medical resources. Endoscopic ultrasonography and laparoscopy can also be used to determine the resectability before surgery, but they are time-consuming, invasive, and expensive. CEA, CA125, and CA19-9 are the most commonly used tumor markers for preoperative diagnosis and postoperative prognosis prediction of cholangiocarcinoma [36, 37]. According to Juntermanns et al. [38], serum CEA and CA19-9 levels are correlated with the stage of cholangiocarcinoma, and patients with higher preoperative CEA and CA19-9 levels tend to have poorer survival and prognosis. Hatzaras et al. [39] reported that a high preoperative serum CA19-9 level often suggests a low survival rate in patients with bile system cancer. However, little is known about the effects of these tumor markers on the resectability of cholangiocarcinoma.

In the present study, we analyzed the resectability of cholangiocarcinoma based on ROC curve analysis. We found that serum CA19-9 is one of the predictors of cholangiocarcinoma, with an AUC of 0.73 and an optimal cut-off of 1064.1 U/mL. Unlike many other previous studies, our results did not rule out the effect of high bilirubin level on CA19-9, mainly for the following two reasons: cholangiocarcinoma is characterized clinically by its insidious onset, and jaundice, as one of the early symptoms of cholangiocarcinoma, is highly suggestive for this disease in most patients [40–42]. Most of our patients presented with jaundice as the first symptom. Therefore, predicting the surgical resectability by analyzing the serum CA19-9 level in patients with jaundice is particularly significant. In contrast, it is believed that serum CA19-9 is mainly affected by tumor severity and serum bilirubin level [37, 43, 44]; however, it is not possible to completely rule out the effect of serum bilirubin and merely analyze the relationship between serum CA19-9 elevation and surgical resection rate in the statistical analysis. The results of our current analysis were more representative of resectability of cholangiocarcinoma. We also analyzed the relationship between bilirubin level and surgical resection rate of cholangiocarcinoma and found that its predictive value was extremely low, suggesting the feasibility of analyzing serum CA19-9 in patients with jaundice. Since CA19-9 is valuable in predicting the resectability of cholangiocarcinoma [45, 46], it can be used as a supplementary tool for preoperative imaging and for comprehensive evaluation of the success rate of an operation, so as to avoid unnecessary surgery.

Notably, serum CA 125 level had a higher correlation with the resectability of cholangiocarcinoma than CA19-9, which may be explained by the fact that CA125 is less affected by bilirubin. In our study, the AUC of CA125 was 0.81 and the optimal cut-off was 17.8 U/mL. Therefore, it is necessary to measure serum CA125 before surgery, together with CA19-9 as an auxiliary marker, to compensate for the defect of preoperative imaging in predicting resectability and to better guide the treatment.

We also analyzed the relationship between serum CEA and the resectability of cholangiocarcinoma. For CEA, the AUC was 0.66, and the optimal cut-off was 2.6 g/mL; the sensitivity of CEA in predicting the resectability was 79.0%, along with a specificity of 48.0%, PPV of 75.5%, and NPV of 53.0%. However, CEA is a broad-spectrum tumor marker and cannot be used as a specific marker for the diagnosis of a malignancy [47, 48]. Therefore, the value of serum CEA in predicting the resectability of cholangiocarcinoma was lower than that of CA19-9 and CA125.

Generally it is not accurate to predict the resectability of cholangiocarcinoma using a single marker [6, 49]. The value of combining three tumor markers in evaluating resectability of cholangiocarcinoma is higher than that of two. Therefore, it is clinically significant for the preoperative detection of tumor markers in patients with cholangiocarcinoma.

On the other hand, tumor markers are supplement preoperative imaging [50]. Comprehensive analysis of clinical manifestations, preoperative imaging findings, and other prognostic factors (including tumor size) can better assess the resectability of cholangiocarcinoma and provide feasible, appropriate, and reasonable treatment for patients [51].

**Figure 4:**
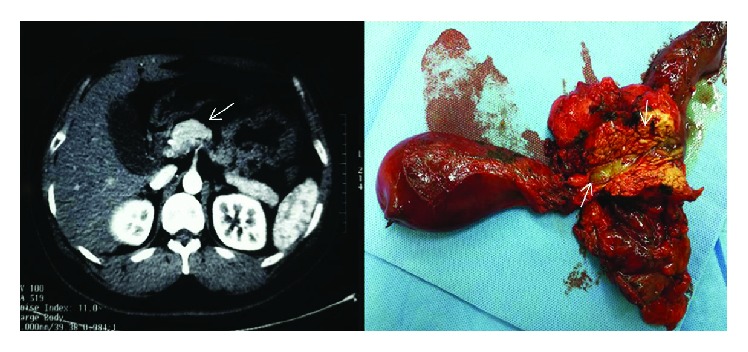
Findings of computed tomography: enhanced computed tomography (CT) revealed a marked high-density area at the distal bile duct (arrowhead). Macroscopic findings: opened bile duct of the resected specimen (arrowhead). Gross appearance of the cut surface of the resected ampulla shows a gray-white tumor measuring 16 mm × 5 mm × 8 mm (arrowhead).

**Figure 5:**
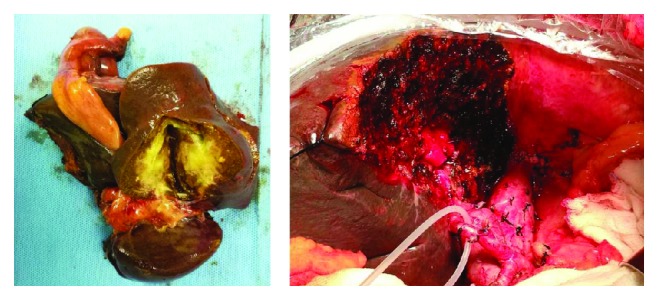
Macroscopic findings: gross appearance of the cut surface of the resected liver shows a gray-white tumor measuring 16 mm × 5 mm × 8 mm.

**Figure 6:**
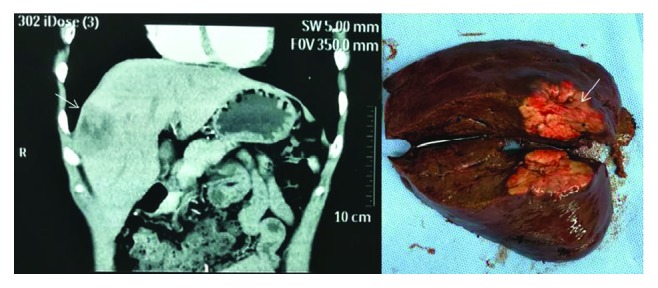
Findings of computed tomography: sagittal plane of computed tomography shows the low-density area (arrowhead) at the right anterior lobe of the liver. Macroscopic findings: gross appearance of the resected liver shows a gray-white tumor measuring 34 mm × 32 mm × 19 mm (arrowhead).

We also apply this combination to clinical practice.  Figure 4 shows one case of distal cholangiocarcinoma. It was difficult for us to know whether the cancer infiltrated the surrounding tissue by preoperative imaging. However, the CA19-9, CA125, and CEA of the patient were lower than the cut-off point in our study. Then radical resection was performed successfully with a negative postoperative pathological margin. Figures 5 and 6 show one case of hilar cholangiocarcinoma and one case of intrahepatic cholangiocarcinoma. It was judged resectable according to our prediction, and the radical resection was performed successfully.

In addition, our retrospective analysis also had limitations in the study design. First, most of the selected patients were diagnosed late and had accompanying hyperbilirubinemia, so it was not possible to accurately analyze the relationship between serum CA19-9 level and resectability of cholangiocarcinoma. Second, our study included patients with jaundice and the results need to be further validated in more comprehensive multicenter studies.

## 5. Conclusions

In conclusion, preoperative serum CEA, CA19-9, and CA125 levels are useful in predicting the resectability of cholangiocarcinoma and may become supplementary diagnostic indicators for evaluating the resectability of cholangiocarcinoma in the future.

## Figures and Tables

**Table 1:** Relationship between clinical features and resectability of cholangiocarcinoma patients.

Features	Resectable (*n* = 50)	Unresectable (*n* = 62)	Total (*n* = 112)	*P* value
Age (yr)	60.9 ± 7.7	63.7 ± 1 0.0	62.5 ± 9.1	
Sex (*n*)				0.172
Men	33	33	66	
Women	17	29	46	
Tumor location (*n*)				0.029
Intrahepatic	4	12	16	
Hilar	12	23	35	
Distal	34	27	61	
Differentiation (*n*)				0.003
High	20	4	24	
Moderate	22	6	28	
Poor	8	12	20	
Tumor diameter (cm)	2.3 ± 0.9	4.5 ± 1.6	3.5 ± 1.3	
AJCC stage (*n*)				0.000
I	2	0	2	
II	41	3	44	
III	7	17	24	
IV	0	42	42	
CEA (ng/mL)	5.0 ± 13.9	19.1 ± 69.2	12.8 ± 52.6	
CA125 (U/mL)	15.3 ± 11.8	48.8 ± 58.7	33.9 ± 47.3	
CA19-9 (U/mL)	257.5 ± 325.6	730.1 ± 527.5	519.1 ± 505.4	
Total bilirubin (*μ*mol/L)	187.1 ± 121.7	219.3 ± 174.9	204.9 ± 153.5	

AJCC Cancer Staging Manual 7th edition. Patients were divided into the resectable and unresectable groups; some patients in the unresectable group were diagnosed according to imaging findings and therefore had no data on pathological stage or AJCC stage.

**Table 2:** Multivariate logistic regression models for predicting the resectability of cholangiocarcinoma.

Observation	Predicted value		
	Radical resection	Nonradical resection	Correct percentage
Radical resection	43	7	86.0%
Nonradical resection	25	37	59.7%
Overall percentage	60.7%	39.3%	71.4%

The accuracy of the model in predicting surgical resectability is higher, reaching 86.0% (*P* < 0.05).

## Data Availability

The data used to support the findings of this study are available from the corresponding author upon request.
